# Sonication versus the conventional method for evaluation of the dental microbiome: a prospective pilot study

**DOI:** 10.1186/s12903-022-02374-0

**Published:** 2022-08-12

**Authors:** Oliver Wagendorf, Peter Menzel, Rolf Schwarzer, Norbert Neckel, Saskia Preissner, Max Heiland, Susanne Nahles

**Affiliations:** 1 Department of Oral and Maxillofacial Surgery, Charité – Universitätsmedizin Berlin, corporate member of Freie Universität Berlin, Humboldt-Universität zu Berlin and Berlin Institute of Health, Charitéplatz 1, 10117 Berlin, Germany; 2 Labor Berlin – Charité Vivantes GmbH, Berlin, Germany

**Keywords:** Microbiome, Tooth extraction, Sonication, Antibiotic resistance

## Abstract

**Objectives:**

To investigate sonication as a new tool in microbiological probing of dental infections.

**Methods:**

Comparison of a standard probing method: intraoperative swab, with sonication, and vortex of the removed tooth, was performed on 20 carious destructed teeth. Illumina high throughput sequencing of the 16S-rRNA-gene was used for assessing the microbial composition. Antibiotic susceptibility has been assigned based on known resistances of each detected species. Probing procedures were compared using Bland–Altmann-Test, and antibiotic susceptibility using the Friedmann-Test and alpha-adjusted post-hoc-analysis.

**Results:**

In total, 60 samples were analysed: 20 intraoperative swabs, 20 vortex fluids, and 20 sonication fluids. Sonication fluid yielded the highest number of bacterial sequencing reads in all three procedures. Comparing the operational taxonomic units (OTUs) of the identified bacteria, significantly more OTUs were found in sonication fluid samples. Phylum and order abundances varied between the three procedures. Significantly more Actinomycetales have been found in sonication fluid samples compared to swab samples. The assigned resistance rates for the identified bacteria (1.79–31.23%) showed no differences between the tested probing procedures. The lowest resistance rates were found for amoxicillin + clavulanate (3.95%) and levofloxacin (3.40%), with the highest in amoxicillin (30.21%) and clindamycin (21.88%).

**Conclusions:**

By using sonication on extracted teeth, it is possible to get a more comprehensive image of the residing microbial flora compared to the standard procedure. If sonication is not available, vortexing is a potential alternative. In immunocompromised patients, especially when actinomycosis is suspected, sonication should be considered for a more detailed microbiological evaluation of the potential disease-causing microbiome. Due to the high rates of antibiotic resistance, a more targeted antibiotic therapy is favourable. Levofloxacin should be considered as a first-line alternative to amoxicillin + clavulanate in patients with an allergy to penicillin.

**Supplementary Information:**

The online version contains supplementary material available at 10.1186/s12903-022-02374-0.

## Introduction

Worldwide, 10% of antibiotic prescriptions are due to dental infections [1]. The most commonly prescribed antibiotics in oral infections are penicillin, followed by lincosamides, macrolides, tetracyclines, and fluoroquinolones [2]. The overprescribing of antibiotics occurs at a rate of 55–80% by dentists [1], leading to an increasing level of bacterial resistance and changes in the composition of the microbiome in odontogenic infections [3–5]. Even though early studies focused on finding one specific microorganism causing dental diseases such as caries, gingivitis, and periodontitis, it is now generally accepted, that dental diseases are caused by a change in the specific surface microbiome of the affected oral tissue, driven forward by multispecies microbial interactions [6, 7].

Taking intraoperative swabs in severe odontogenic infections [8] to verify microbiological and antibiotic susceptibility of the present bacteria to ensure a targeted antibiotic regime is the usual procedure. Due to contamination or growth of too many different species or subspecies, which are hard to cultivate, the outcome of this method is poor and calls for better alternatives for microbiological sampling [9–12].

It is known that the composition and structure of the oral microbiota differ between the existing oral niches [13]. Testing of the saliva reveals microorganisms from various oral niches, but studies have demonstrated that it will not represent the entire oral microbiome [14]. However, tooth surfaces provide an ideal environment for bacterial growth and formation of dental plaque, so they will represent a higher microbial richness and diversity [15]. Besides preventing biofilm-related infections, the analysis of the sensitivity, specificity, and amount of the bacteria in the microbiome plays a decisive role in a targeted antibiotic regimen [10].

Bio-film-related infections do not only occur in the mouth but also in different regions of the human body. One such type of infection associated with biofilm formation is prosthetic joint infections (PJIs). PJIs are the second most common cause of prosthetic joint failure [16] and it is known that they are also caused by bacteria forming an organised biofilm on the implant surface, as oral bacteria do on teeth [17].

Orthopaedic studies have published data about the special procedure of sonication [16, 18]. Thus, the sensitivity and specificity of using the sonication fluid of explanted prostheses for detecting bacteria were shown to be increased compared to the normal procedure, such as synovial fluid cultures and tissue samples or swabs [18]. The number of bacteria identified was up to 10,000 times higher than in standard procedures [18].

Furthermore, sonication was used as an established method on breast, urinary, endovascular and cerebral implants [19–22]. Less is known about the use of sonication for microbiological testing in dentistry. It was mostly used to detach the biofilm from dentures, to evaluate the amount of *Candida* species [23], the microbiome of dentures in relation to denture stomatitis [24], and in vitro, to detach the formed biofilm from carbon or titanium surfaces [25] for analysis of the formed biofilm in relation to the implant surface. Regarding the orthopaedic and dental results, the sonication method promises improved detachment of the biofilm located on the teeth, resulting in a complete image of the potential disease-causing microbiome. In these previously published studies, only the sonication method was used to identify the bacterial colonisation process without comparable methods. Furthermore, Almaguer-Flores et al. could demonstrate the strong influence of chemical and physical properties of the substrate in the colonisation of oral bacteria (17).

To the best of our knowledge, no data are available to compare the sonication process to the conventional swab method for microbial investigation. Even in compromised patients, the safe and sufficient extraction of material for microbiological testing and therefore a better knowledge about the predominant microorganisms could lead to a more targeted approach in antibiotic treatment.

Therefore, the aim of the present study was to evaluate the outcome of bacterial DNA extraction and 16S-rRNA amplicon sequencing in the sonication fluid compared to vortex fluid and the standard method, intraoperative swab of the alveolus.

## Methods

### Inclusion criteria and surgical procedure

The research proposal for this prospective study was approved by the Ethics Committee of the Charité Universitätsmedizin Berlin, Germany (EA4/194/19) and complies with the STROBE guidelines. It conforms to the Declaration of Helsinki and the European Medicines Agency Guidelines for Good Clinical Practice. The study’s inclusion criteria were age of majority, at least one permanent tooth, which had to be removed due to carious destruction, no abscess, no systemic disease or drugs, and no nicotine abuses.

Twenty healthy patients, twelve men (mean age 54.2 years) and eight women (mean age 61.9 years) with at least one non-restorable premolar in the mandible were included in the study. All teeth were removed due to carious destruction.

All surgical procedures were performed under sterile conditions by one experienced surgeon under local anaesthesia. Extraction was performed atraumatically, using forceps and elevators. After the removal of the tooth, the extracted tooth was directly placed in a Falcon tube filled with 4 ml Urine Conditioning Buffer™ (UCB™, Zymo Research Corp, CA 92614, USA). Thereafter, a swab, using “DNA/RNA Shield Collection Tube w/ Swab” (Zymo Research Crop, CA 92614, USA), was taken from the extraction socket, representing the standard procedure of microbiological sampling.

All samples have been stored in the refrigerator after collection.

### Microbiological preparation and assessment

All samples were transferred to the microbiological laboratory within 24 h, where the probes were processed on a laminar flow bench (Safety cabinet, Thermo Scientific, Langenselbold, Germany). The Falcon tube was vortexed for 30 s. Afterwards, 2 ml of the fluid was removed and placed in a second Falcon tube for further evaluation. Sonication was performed for 1 min at 40 kHz (BactoSonic, Bandelin electronic, Berlin, Germany), following vortexing for another 30 s, as already established in the sonication of endoprosthesis [26]. The swab and the two Falcon tubes per patient, containing the sonication fluid and vortex fluid, were transferred to the microbiology laboratory for analysis. For each procedure, one control sample (swab without probing, vortex, and sonication without tooth) was taken and analysed separately, to evaluate the kit-specific microbiome. Total processing of all samples was performed by the same person.

### Microbial analysis

DNA was extracted and purified into 50 µl elution buffer using the DNeasy PowerSoil Pro-Kit (Qiagen). The 16S-V3-V4-PCR was performed using UCP-Multiplex-PCR Mastermix (Qiagen) according to the 16S Metagenomic Sequencing Library Preparation Protocol (Illumina, Primer: Fwd = CCTACGGGNGGCWGCAG, Rev = GACTACHVGGGTATCTAATCC) with 2 μl DNA [27]. In a subsequent PCR, index sequences were added to the purified PCR product. Samples were pooled in one sequencing library and sequenced on an Illumina MiSeq with v2-reagents in 2 × 250 bp paired-end reads with a mean sequencing depth of > 100,000 reads per sample. The number of reads per sample serves as a rough estimation of the amount of bacterial DNA.

After sequencing, paired reads of each amplicon were merged and clustered to Operational Taxonomic Units—OTUs, using the usearch package by clustering identical sequences with an identity of at least 97%. A representative consensus sequence was assigned to every OTU and OTUs were quantified by counting the number of reads mapped to each OTU consensus. The consensus sequences of each OTU were compared to the NCBI 16S-Microbial and NT Reference-Database, using NCBI BLAST (megablast). The OTUs were taxonomically classified based on the best database match (with a minimum identity of 97%) [28]. If multiple database hits matched the OTU sequence with the same identity the OTU was classified as the lowest common ancestor of the different database hits. If no match was found the respective OTU was labeled as unclassified. The reason for the alignment to more than one species relates to the fact that the V3-V4 region in different species can be identical.

Antibiotic susceptibility was evaluated by using “Antibiotics in Laboratory Medicine, 6th Edition” [29]. All found bacterial species were checked for existing enzymes or intrinsic resistances, which are able to inactivate the antibiotics used in clinical routine. Susceptibility was evaluated for amoxicillin, amoxicillin + clavulanate, clindamycin, doxycycline, and levofloxacin. For each sample, we calculated the percentage of bacterial species that have known resistances to any of the 4 antibiotics groups.

### Statistics

Statistical analysis was performed using “R” and MathCalc version 15.8, Graphs were created using “Phyton” and MathCalc version 15.8. Overall, 60 different probes, three for each patient, were analysed. Descriptive Statistics were performed for a number of reads, diversity, phyla distribution, and identified taxa for all samples. Bland–Altmann-test was used to check for significant differences over all three procedures. Friedmann-Test was used to check for differences in antibiotic resistance. Post-hoc analysis was alpha-adjusted and performed to check for significant differences between specific antibiotics. A P-value < 0.05 was considered statistically significant.

## Results

Of the 60 samples, the mean number of sequenced reads for each procedure, was 142,858 in the swab, 180,739 in the vortex, and 217,972 in the sonication. The diversity of all samples was evaluated by counting the number of OTUs. Each OTU symbolised one specific bacterial entity. 253 different species were detected in all samples.

In total OTUs, the swab compared to sonication showed a significant difference (p = 0.04; 95% CI − 22.19 to − 0.51). A negative confidence interval highlighted that sonication produced a significantly higher number of OTUs compared to the swab.

**Fig. 1:**
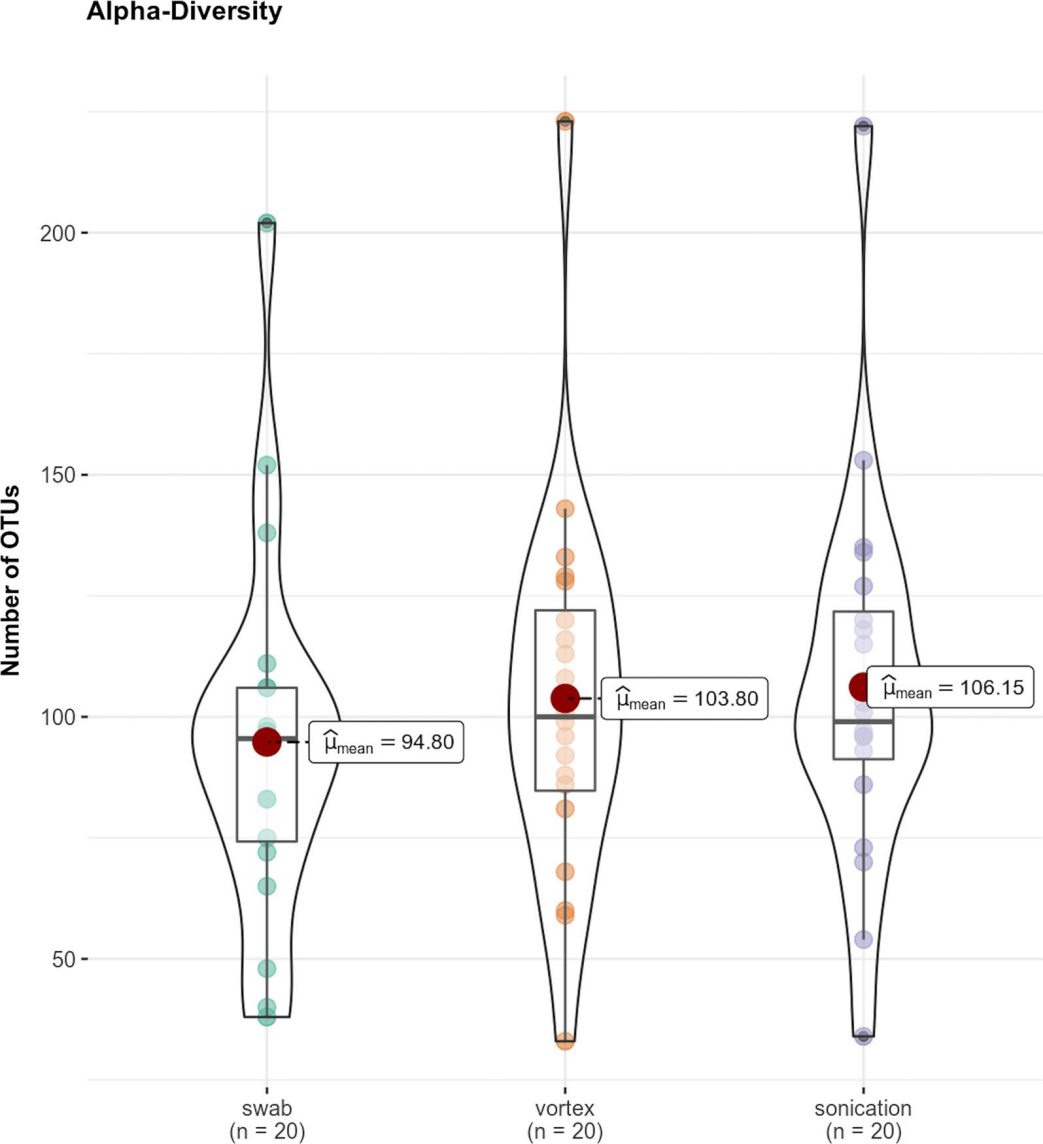
Mean OTUs in swab, vortex, sonication

**Fig. 2:**
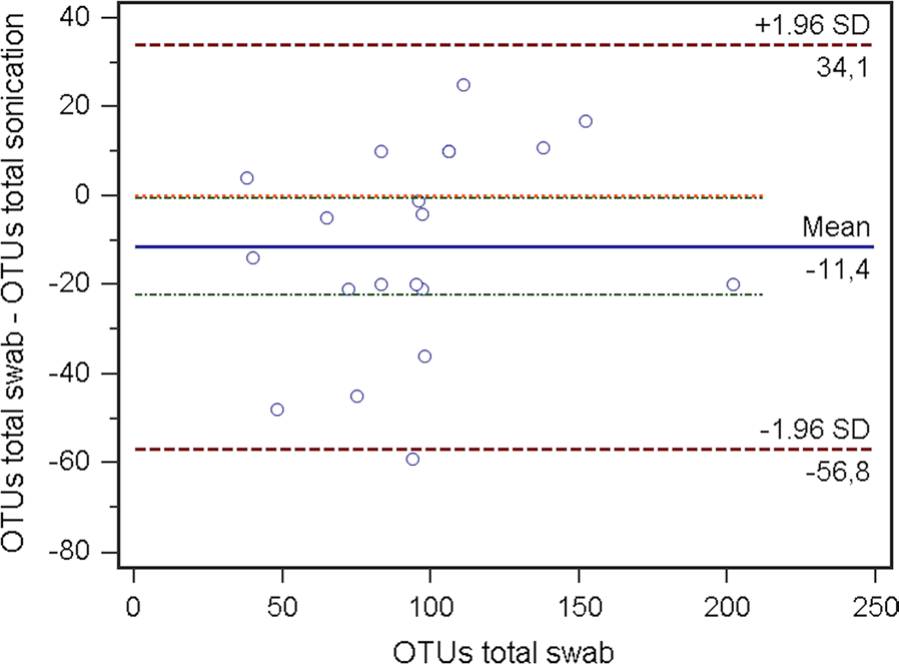
Bland–Altman-Plot for comparison swab and sonication, regarding found OTU

All other combinations showed no significant difference. The mean number of OTUs is shown in Fig. 1. Bland–Altman-Plot for comparison swab and sonication, regarding found OTUs is shown in Fig. 2.

**Fig. 3:**
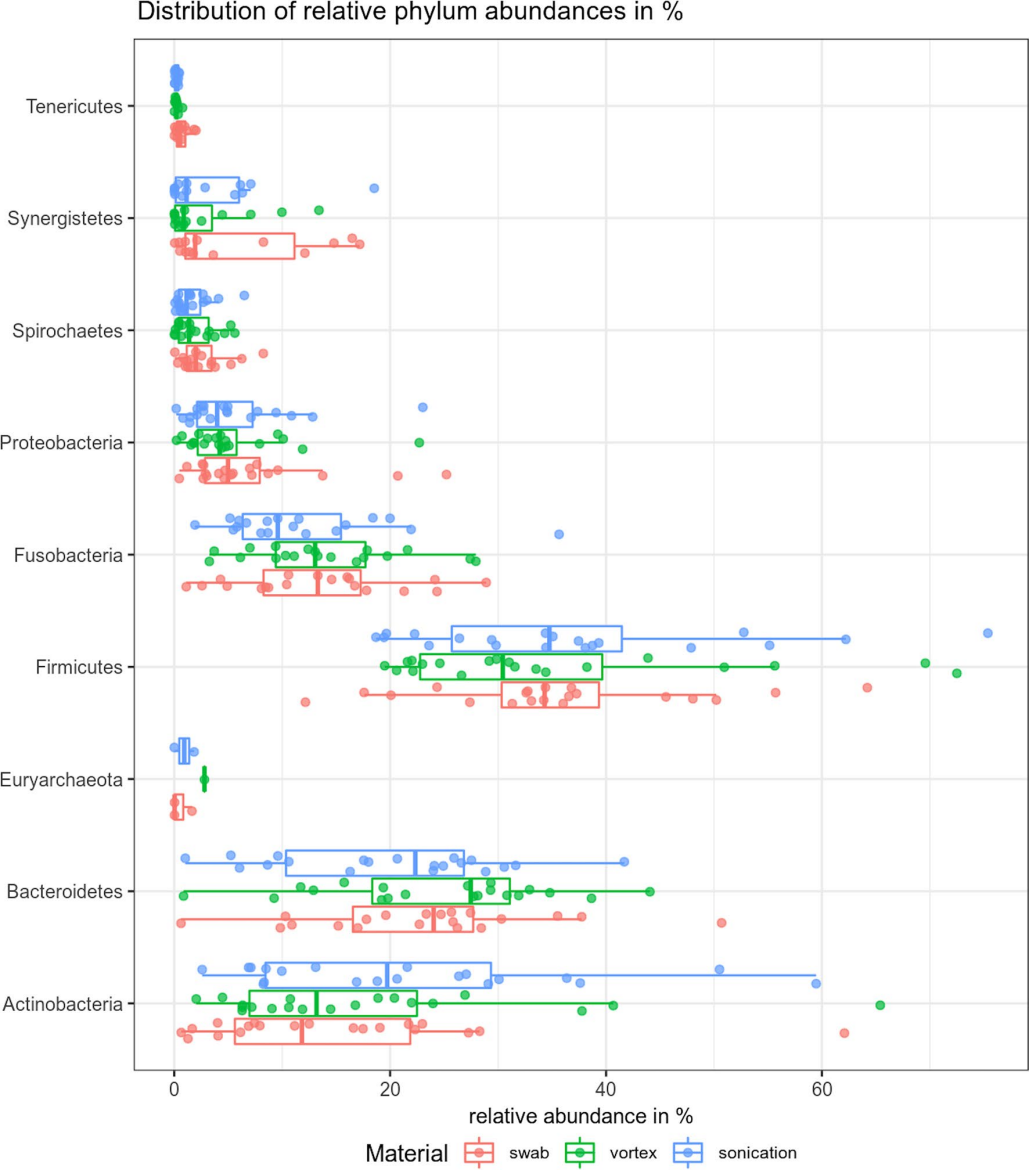
Distribution of relative phylum abundance in percentage

In Fig. 3, the distribution of relative phylum abundances is shown.

There is a high variance in relative phylum abundance in all three sampling procedures. In Actinobacteria (p < 0.01), Bacteroidetes (p < 0.01), and Tenericutes (p < 0.0396), significant differences between swabs and sonication could be found. The confidence interval in Actinobacteria (− 11.19 to − 2.57) showed a higher percentage in sonication in contrast to Bacteroidetes (0.86 to 5.18) and Tenericutes (0.01 to 0.56), where a higher percentage of appearance was found in the swab. Only slight tendencies towards a higher amount of Actinobacteria could be found in vortex, compared to swabs.

On Order-Level, as shown in the supplements, Actinomycetales (p < 0.01), Bacteroidales (p < 0.01), Corynebacteriales (p < 0.01), Flavobacteriales (p = 0.03), and Mycoplasmatales (p = 0.04) showed significant differences when comparing the swab to the sonication procedure (Additional file 1). In Actinomycetales (confidence-interval − 7.29 to − 2.39), Corynebacteriales (95% CI − 2.09 to − 0.34), and Flavobacteriales (95% CI − 2.32 to − 0.16), higher percentage abundances could be found in sonication, in contrast to Bacteroidales (95% CI 2.0777–6.4547) and Mycoplasmatales (95% CI 0.015–0.56) where a higher relative abundance was found in the swab compared to sonication (Additional file 2).

For each experimental group, the mean fraction of bacterial species with intrinsic resistance against certain antibiotics are shown in Table 1.

**Table 1:** Mean resistance among swab, vortex, and sonication in percentage point

Resistance to	Swab (%)	Vortex (%)	Sonication (%)
Amoxicillin	27.12	32.29	31.23
Amoxicillin + Clavulanate	6.51	2.78	2.56
Clindamycin	19.48	24.43	21.74
Doxycycline	17.75	20.56	20.48
Levofloxacin	4.58	1.79	3.82

Mean resistance among swab, vortex and sonication in percentage point

In sonication fluid, vortex fluid, and swab, no significant differences were found in the resistance rate of the bacteria.

A significant difference (p < 0.01) could be found in bacterial resistance to different antibiotics using Friedmann-Test. Assigned ranks were 1.58, 1.73, 3.35, 3.60, and 4.75 for amoxicillin-clavulanate, levofloxacin, doxycycline, clindamycin, and amoxicillin, respectively, representing amoxicillin-clavulanate with the lowest and amoxicillin with the highest resistance percentage rate. The post-hoc test for differences in specific antibiotic resistance between each analysed individual showed significant differences in all combinations except for amoxicillin-clavulanate with levofloxacin and clindamycin with doxycycline. P-values for each comparison are shown in Table 2.

**Table 2:** Antibiotic resistance comparison

	Comparison									
	Amoxicillin-clavulanate	Clindamycin	Doxycycline	Levofloxacin	Clindamycin	Doxycycline	Levofloxacin	Doxycycline	Levofloxacin	Levofloxacin
	Amoxicillin	Amoxicillin	Amoxicillin	Amoxicillin	Amoxicillin-Clavulanate	Amoxicillin-Clavulanate	Amoxicillin-Clavulanate	Clindamycin	Clindamycin	Doxycycline
p-Value	0.000	0.000	0.001	0.000	0.000	0.001	0.877	0.232	0.000	0.001

Antibiotic resistance comparison

No relevant delay in microbiological results could be observed between the tested procedures.

## Discussion

To summarise the present results, by sonicating the tooth, significantly more bacteria could be detected compared to the swab, as shown by the higher number of OTUs in sonication samples. Furthermore, even the microbial composition of the analysed samples differed between the tested procedures. Moreover, it was possible to find some bacteria which could not be found in the standard procedure for microbiological testing. Focusing on antibiotic resistance rate, no significant difference between sonication fluid, vortex fluid, or swab could be found. A significant difference could be shown in the comparison of the resistance rate of the evaluated antibiotics.

The knowledge of the bacterial composition is crucial for a targeted and effective antibiotic regime. Next-generation sequencing using 16S rRNA gene has shown good results for identification of the oral microbiome [30–35]. The microbial composition reported is similar to earlier studies focusing on the oral bacterial composition [30, 32, 33, 36]. The most commonly used sample types to study the healthy oral microbiome and its changes in various diseases were saliva, oral rinse, or niche-specific samples, e.g. supra- or subgingival plaque or tongue swab [31]. Whole teeth have never been analysed before.

For evaluation of the purity of the collected specimen, one control sample for every procedure was taken and analysed separately, as recommended by Zaura et al. [31]. The results showed a low number of reads and diversity, confirming negligible contamination due to the kit-specific microbiome.

The number of reads is a semi-quantitative tool. It is highly affected by the number of PCR cycles performed, the taxa identified, and the sequencing runs itself [37, 38]. Therefore, no precise quantitative measurement is possible regarding how many times more DNA can be found in the sonication fluid compared to the swab or vortex.

In the present study, all patient samples were located on the same sequencing run, with the same number of cycles and same primer respectively, which provides comparability between the swab, vortex, and sonication fluid (Additional file 3).

Taking all of this into consideration, it is highly likely that there is a higher amount of DNA in the sonication fluid compared to the swab and vortex in each patient.

The procedural difference between sonication in contrast to the swab is that they are performed on extracted teeth, resulting in biofilm loosening on the whole surface, whereas the swab was only taken from the alveolus. Therefore, the quantity of the material gathered is probably higher and might have a distorting effect.

The composition of each oral microbiome is different, not only in the number of reads but also in the taxa found. A possible reason for this could be the diversity of the different microbiome surfaces and inter- and intra-individual variations [39]. The oral biofilm development over time is a complex interaction of different species which colonise oral surfaces to form an organised multispecies community with a specific composition. This is caused by the different prevailing physical and biological conditions in the oral habitat, such as surface texture, cell desquamation, or aerobic capacity in the specific niche [6]. Faust et al. demonstrated that the microbiome in different types of samples is similar but nevertheless different [40], highlighting that the source of sampling is crucial for proper microbial testing and especially so for antimicrobial susceptibility testing. This difference could be a reason for the differing microbiological results of the swab and vortex or sonication fluid. Especially regarding the aerophilic capacity of each bacterium, a more anaerobic bacterial composition should be expected in the alveolus or the periodontal pocket than on the tooth surface. By comparing the whole tooth surface, symbolised in the sonication fluid, the bacterial distribution is expected to be different from the bacterial distribution of the alveolus itself.

Apart from this, the contamination of sample extraction kits, during production, can have a potentially misleading impact on the microbiome analysis and consequent conclusion [41].

Even though the biofilm disruption on the whole surface of the tooth could be a potential bias, every bacterium located on the tooth could be able to cause further or could be the reason for the specific infection. Therefore, this setup resembles the reality of biofilm behaviour in the extraction setting in which the potential dissemination of parts of the biofilm can occur, resulting in severe consequences such as infectious endocarditis [42].

A potential criticism could be the amplification of the 16S rRNA gene. Using this procedure, there is no differentiation between bacteria, dead or alive.

Nonetheless, detection of dead bacteria is a potential benefit, because the procedure of swab taking negatively affects the viability of anaerobic bacteria [9]. In normal culture-based analysis, only living bacteria can be examined.

So, the procedure of sonication could be a further influencing factor and cause potential bias. During sonication, little air bubbles explode on the surface of the tooth, leading to the loosening of the biofilm. Bacteria, which are anaerobes or facultative anaerobes, will be highly affected by this excess oxygen [43], even though it could be shown that these bacteria are still alive after sonication of endoprosthesis [18]. Using the 16S rRNA gene, this correlation can be ignored, due to the stability of the genome, even when the bacteria are compromised.

Focusing on the study design, neither orthopaedic nor else studies, which are comparing direct 16S-rRNA-gene analysis and the difference in the microbiome distribution in different sampling modalities exist. Only a few studies have been investigating, whether using 16S-rRNA-gene analysis resulted in similar or even improved results, than normal microbiological testing [26, 44–46]. Due to this fact and the appropriate results of 16S-rRNA-gene analysis in microbiome analysis of the oral cavity, we assume, that this is a reliable tool for such investigation [30, 32, 34, 47]. Also in settings, where a dental infection is a potential causing of more severe disease such as medication-related osteonecrosis of the jaw, this sampling method is a potential tool to evaluate and identify the disease-causing bacteria, also in areas hard to reach or where contamination of the normal probing method, swab or tissue sample, is to be expected.

The present results revealed that the amount of Actinomycetales is underrepresented in the normal probing procedure. One potential life-threatening disease, which is hard to diagnose, is craniofacial actinomycosis [48]. In most cases, it is associated with odontogenic infections [49]. Therefore, it can be assumed, that by only taking a swab in combination with normal microbial culture, there is a general underestimation of this disease. Especially in patients undergoing or following radiotherapy due to head and neck cancer, this disease is a feared complication [48]. Also, in medication-related osteonecrosis of the jaw, Actinomyces spp. seem to play a major role in disease progression [50–52].

Focusing on Bacteroidales and Mycoplasmatales, which had a higher abundance in swabs, the read numbers of these orders were higher in sonication than in the swab. Showing that there were no bacteria missing but because of the higher amount of Actinomycetales, the relative abundance was lower than in the swab.

The antimicrobial susceptibility to specific antibiotics was not tested in bacterial culture or by molecular genetic analysis, which is a downside of this study. Nevertheless, it was not the primary objective of this investigation to precisely analyse antibiotic resistances, but rather to evaluate the procedure of sonication or vortex as a new tool in microbiological testing in oral surgery. Due to this limitation, no exact statement regarding proper antibiotic treatment can be made.

Heim et al. have already shown the increasing level of resistance found in cultures [53]. Literature-based resistances show the same percentage of resistance as that assigned by enzyme-based evaluation. In particular, the anaerobic species, which are hard to cultivate, show a higher resistance to clindamycin [54], which is still the antibiotic of choice in penicillin-allergic patients [2, 55] according to the German Guidelines [8]. By also evaluating doxycycline and levofloxacin, it could be shown that the resistance rate of amoxicillin with clavulanate was similar to levofloxacin and clindamycin to doxycycline.

Focusing on the analysed resistance rates, a change to the use of levofloxacin as a first-line alternative in severe cases of odontogenic infections should be considered in patients with an allergy to penicillin. Levofloxacin is similar to moxifloxacin, which is already in use in odontogenic infections [2]. In 2011, a comparison of clindamycin and moxifloxacin showed similar outcomes, but with a lower rate of adverse effects in moxifloxacin [56]. Taking this into consideration, levofloxacin or moxifloxacin should replace clindamycin as the first line alternative to amoxicillin with clavulanate in severe odontogenic infections. Due to the high rates of overprescribing of dental antibiotics [1], this change should be exclusively provided for hospitalised patients. The inpatient setting is also preferable for fluoroquinolones, due to their possible side effects like increasing the QT-interval [55]. Other possible side effects are tendinopathy, especially with long-term use [57], and drug-drug interactions. Because of the chondrotoxicity, it is not recommended to use this during pregnancy and in children.

In conclusion with the help of sonication, it was possible to find additional species which were not found in the standard procedure, swabs. The whole microbiome constitution differs, showing a potential incongruence between the standard method and our shown procedure. Still, the microbiomes found were similar in the swab, vortex, and sonication. Consequently, in high-risk patients requiring a more targeted antibiotic treatment (e.g., complex infections, former or ongoing radiotherapy, former or ongoing bisphosphonate medication, congenital heart disease, or immunosuppression) sonication of the tooth should be considered to gain a more complete image of the potential disease-causing microbiome. This sample provides the option to obtain as much information on the bacterial colonisation of the tooth as currently possible, meaning that it can therefore improve treatment as well as clinical outcomes. Early targeted treatment or the prevention of severe complications in high-risk patients can be necessary for their survival.

### Limitations

This evaluation was performed on healthy patients, where the hosts’ anti-infective capability is high and severe complications are rare. Due to the small cohort and the characteristic as a pilot study, further investigation should be performed not only focusing on the differences between sonication and the standard procedure for microbial testing in the treatment of infections of the maxillo-facial region. Additionally, the focus should be laid on changes in the oral microbiome in immune-compromised patients. No differentiation has been performed regarding the location of the teeth or the grade of carious destruction.

Yet, sonication could be a tool, especially for immune-incompetent patients, to improve the overview of the bacteria in the infected area, allowing for a more targeted antimicrobial therapy. Also, differences in conventional microbiological testing: bacterial culture, identification, and bacterial susceptibility are planned, to validate the found data, in a bigger cohort. If sonication is not accessible, we could show that vortex could also be considered for loosening of the biofilm on extracted teeth.

## Supplementary Information

**Additional file 1.** Distribution of relative order abundances in %.

**Additional file 2.** Differences in percentage in relative order abundance in each procedure.

**Additional file 3.** Rarefraction curves for every patient.

## Data Availability

The datasets used and/or analysed during the current study are available from the corresponding author upon reasonable request.
