# Supplementary figures and images for: Sonication versus the conventional method for evaluation of the dental microbiome: a prospective pilot study
Source: BMC Oral Health. 2022 Aug 12;22:348. doi: 10.1186/s12903-022-02374-0 (PMC9375313; doi:10.1186/s12903-022-02374-0)

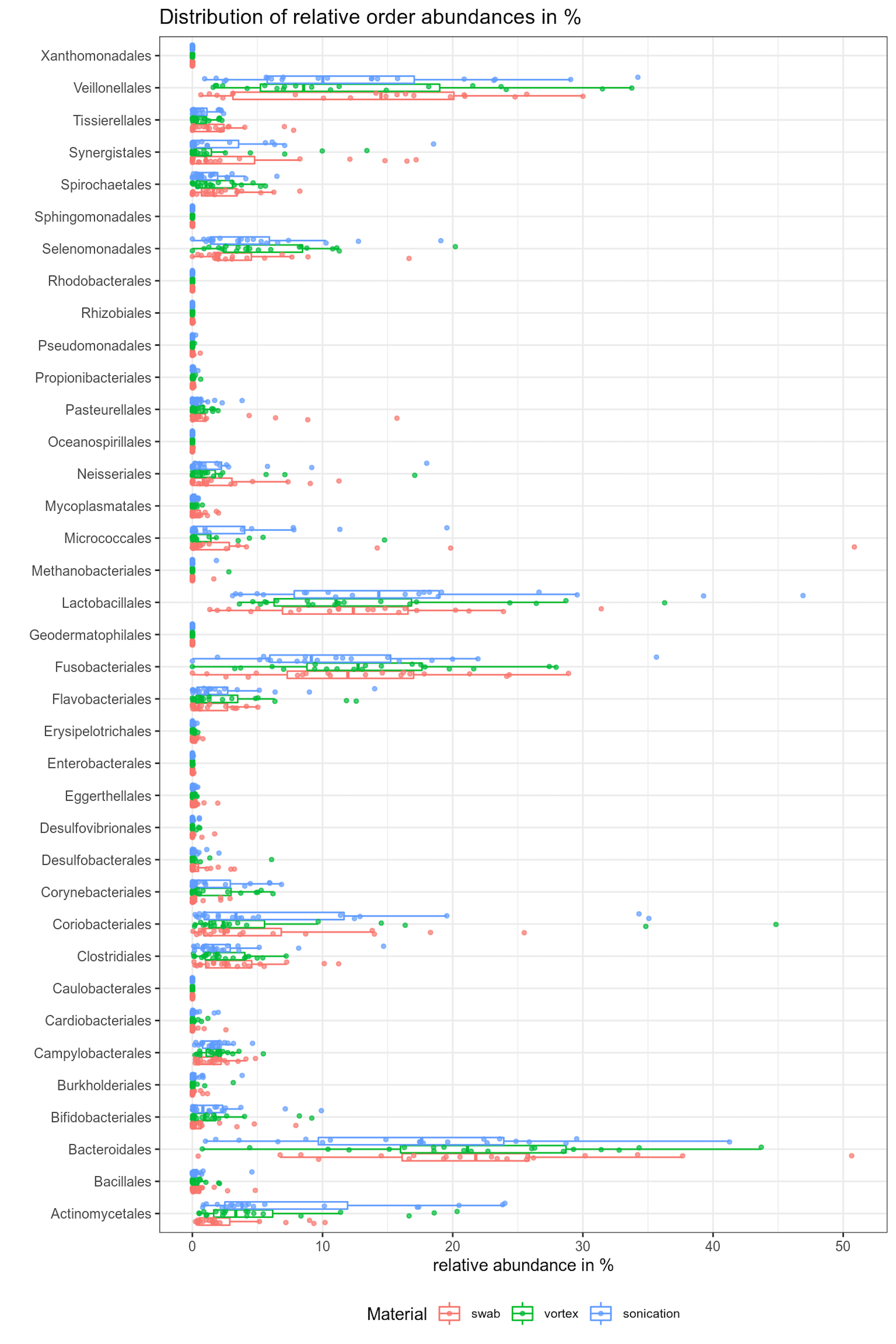

Supplement: Supplementary file 1 — Additional file 1. Distribution of relative order abundances in %. [file 12903_2022_2374_MOESM1_ESM.jpg]

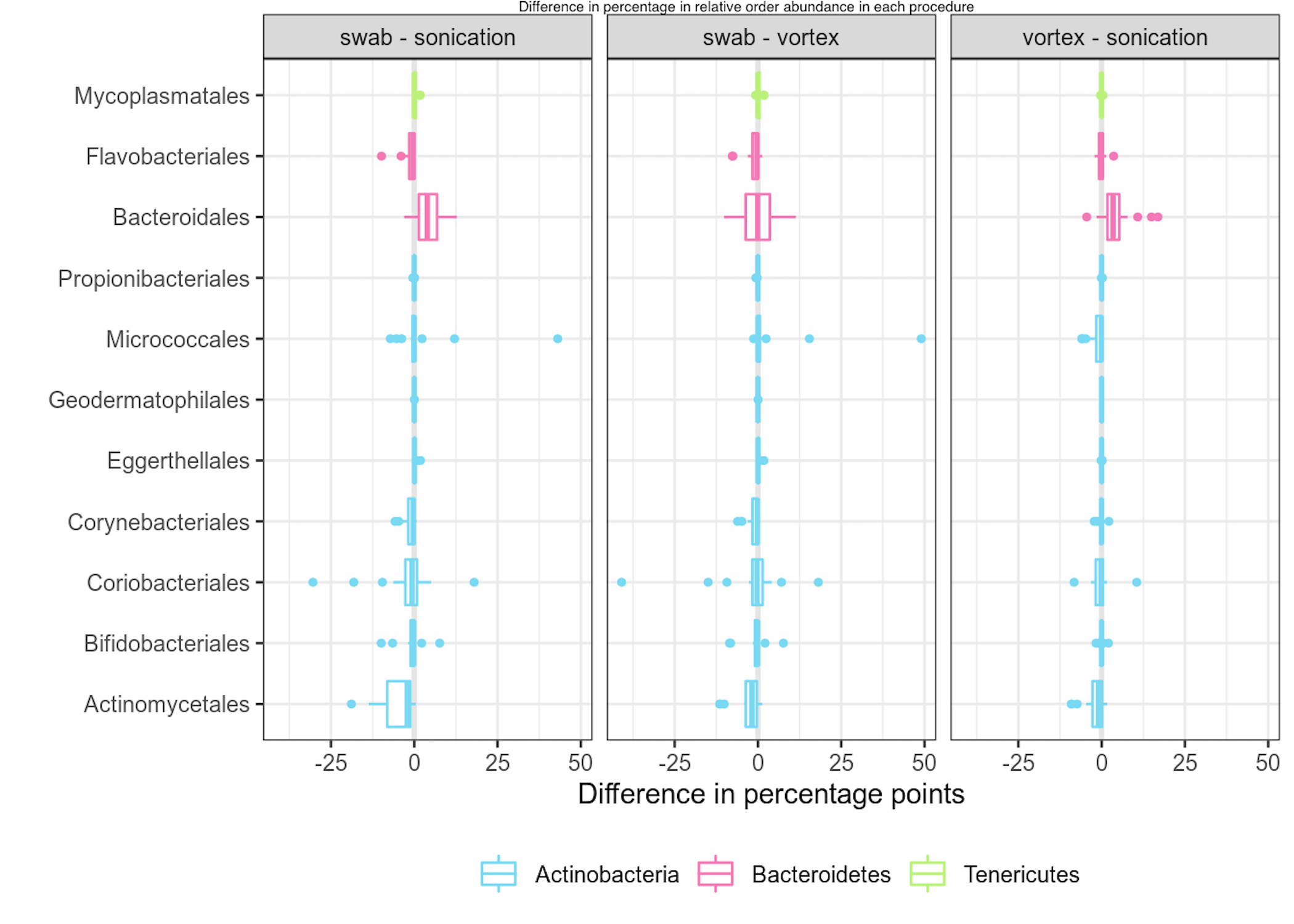

Supplement: Supplementary file 2 — Additional file 2. Differences in percentage in relative order abundance in each procedure. [file 12903_2022_2374_MOESM2_ESM.jpg]

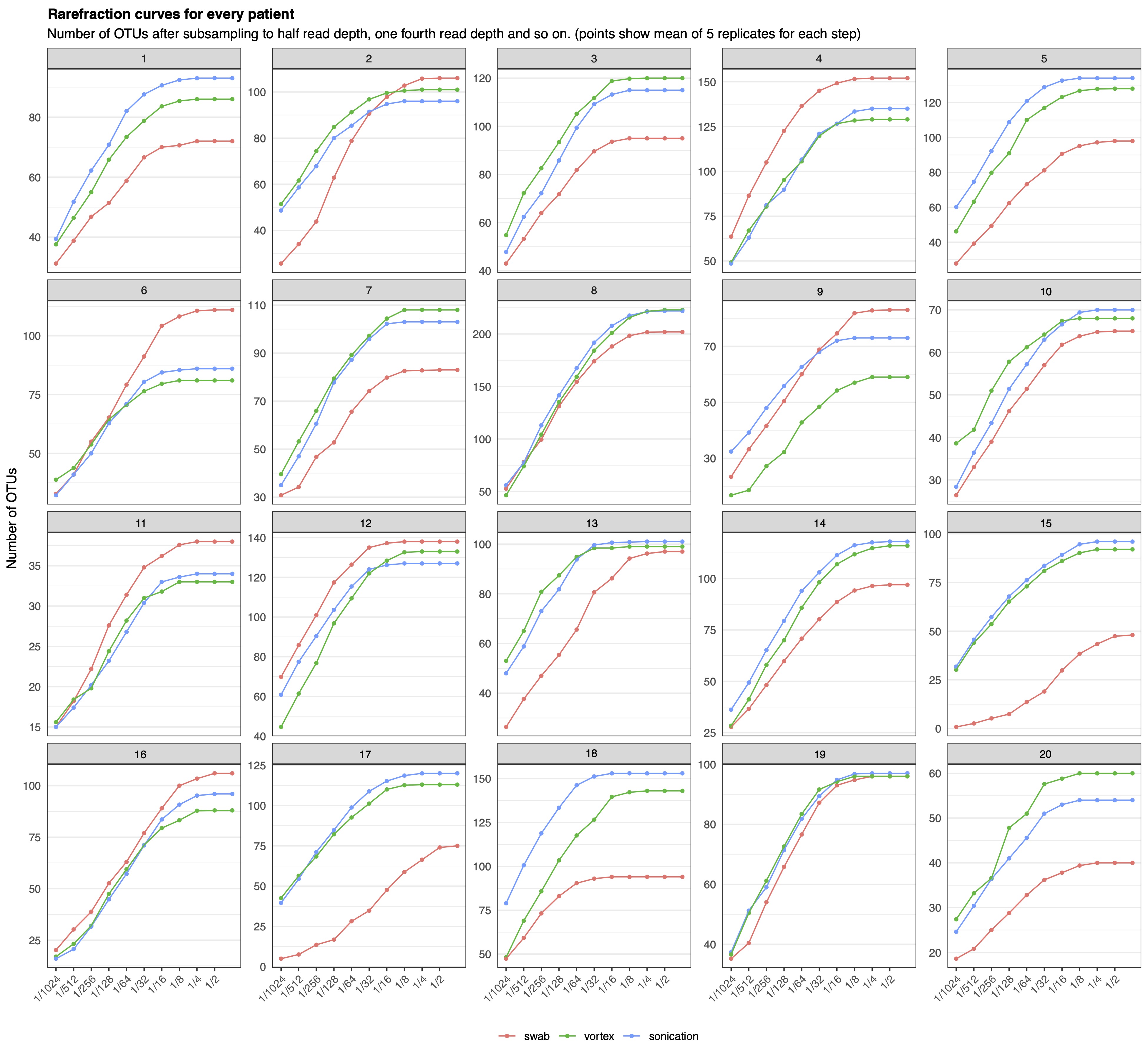

Supplement: Supplementary file 3 — Additional file 3. Rarefraction curves for every patient. [file 12903_2022_2374_MOESM3_ESM.jpg]
